# MONICET: The Azores whale watching contribution to cetacean monitoring

**DOI:** 10.3897/BDJ.11.e106991

**Published:** 2023-08-08

**Authors:** Laura González García, Marc Fernández, José M. N. Azevedo

**Affiliations:** 1 Institute of Marine Sciences - OKEANOS, University of the Azores, Rua Professor Doutor Frederico Machado 4, 9901-862, Horta, Portugal Institute of Marine Sciences - OKEANOS, University of the Azores, Rua Professor Doutor Frederico Machado 4, 9901-862 Horta Portugal; 2 MARE – Marine and Environmental Sciences Centre/ARNET - Aquatic Research Network, Agência Regional para o Desenvolvimento da Investigação Tecnologia e Inovação (ARDITI), Funchal, Portugal MARE – Marine and Environmental Sciences Centre/ARNET - Aquatic Research Network, Agência Regional para o Desenvolvimento da Investigação Tecnologia e Inovação (ARDITI) Funchal Portugal

**Keywords:** citizen science, long term data series, opportunistic data, marine mammals, turtles

## Abstract

**Background:**

The Azores islands have been historically linked to cetaceans, becoming an example of a successful transition from whaling to whale watching. Twenty-eight cetacean species have been sighted in these waters, making the archipelago one of the most recognised whale and dolphin watching destinations worldwide. The business is well-established in the region, operates in four of the nine islands year-round or seasonally and provides an excellent opportunity to collect long term information on cetacean distribution and abundance in an affordable way. Continuous monitoring is indeed essential to establish baseline knowledge and to evaluate cetacean response to potential natural or anthropogenic impacts. Opportunistic data greatly complement traditional dedicated surveys, providing additional support for appropriate management plans.

**New information:**

The MONICET platform has been running continuously since 2009 as a collaborative instrument to collect, store, organise and disseminate cetacean data voluntarily collected by whale watching companies in the Azores. In the period covered by this dataset (2009-2020), 11 whale watching companies have voluntarily provided data from the four islands of the archipelago where whale watching takes place. The dataset contains more than 37,000 sightings of 25 species (22 cetaceans and three turtles). This manuscript presents the first long-term whale watching cetacean occurrence dataset openly available for the Azores. We explain the methodology used for data collection and address the potential biases and limitations inherent to the opportunistic nature of the dataset to maximise its usability by external users.

## Introduction

Collecting long-term wildlife ecological data is challenging, especially for highly mobile species such as cetaceans which, in addition, inhabit remote regions like the high seas (Redfern et al. 2006, Fernandez et al. 2022). Cetaceans are considered ocean sentinels, in the sense that information about their ecology and distribution (e.g. changes in the phenology of migrations, shifts, expansions or reductions of their distribution ranges or changes in relative abundance over time) can be linked with the health of marine ecosystems (Moore 2008). Now more than ever, long-term ecological data are needed to address major issues of current environmental concern whose temporal scales span over decades and have global impacts. In fact, the impacts of global warming, pollution or overexploitation can only be adequately addressed in the long term (Jones and Driscoll 2022). For these reasons, EU Member States are required by the Marine Strategy Framework Directive (Directive 2008/56/CE) to monitor the status of their marine biodiversity in order to ensure a Good Environmental Status. However, dedicated cetacean surveys do not address these needs because, although they provide high-quality information, it is limited in both space and time by the complex and expensive logistics involved (Evans and Hammond 2004). In this context, commercial activities and citizen science have emerged as new opportunities for data collection, providing cost-effective access to long-term information that otherwise would be unavailable (Devictor et al. 2010, Theobald et al. 2015, Brown and Williams 2019).

The flagship status and charismatic nature of whales and dolphins have been widely recognised, making them ideal for maintaining the engagement of citizens in science and conservation. The attraction of these species has been demonstrated by the worldwide increase in whale watching over the last decades. The rapid expansion of this business has provided an educational tool for the public and an excellent opportunity for science (Dickinson et al. 2012, Higham et al. 2014, Currie et al. 2018a). In fact, whale-watching vessels have become opportunistic platforms of observation for citizen-science programmes, collecting data over long periods on a specific area. This results in a cost-effective survey method that allows the collection of precious cetacean information, which would likely be inaccessible without this collaboration and complements traditional surveys, particularly in areas with no baseline information or where funding is limited or not available (Currie et al. 2018a, Fernandez et al. 2018, Callaghan et al. 2021). Several monitoring programmes from opportunistic platforms have proven effective for cetacean data collection at sea, such as ferry or cargo routes or regular touristic trips. Successful examples include the ORCA Survey Network off the UK or the Fixed Line Transect Mediterranean monitoring Network working from European Atlantic and Mediterranean ferries, respectively (Kiszka et al. 2007, Arcangeli et al. 2012, Tepsich et al. 2020); the CETUS project, monitoring from cargo boats from Macaronesia and mainland Portugal (Correia et al. 2019); or the Kakila database, compiled from different whale watching sources in the West Indies (Coché et al. 2021). Opportunistic data obtained from whale watching have been used in multiple scientific publications to address, for example, cetacean distribution (Vinding et al. 2015, Alves et al. 2018), habitat preferences (Moura et al. 2012, González García et al. 2018, Fernandez et al. 2021, González García et al. 2022b) or animal movements (Currie et al. 2018b, Mullin et al. 2022).

The mid-Atlantic archipelago of the Azores has been linked to cetaceans since its colonisation in the 15^th^ century. The first evidence of interaction refers to locals benefitting from stranded carcasses or animals found dead at sea (Azevedo 1991). In the mid-18^th^ century, north American whalers arrived on the islands looking for new whaling grounds for sperm whales *(Physetermacrocephalus)* (see review in Prieto et al. (2013)). Initially, Azoreans were recruited as crew; and soon, the American whaling technique was adapted to a shore-based industry that expanded through all the islands. Land observers were responsible for finding the whales and guiding small open boats to them. Sperm whales were hunted primarily because of their oil, which was progressively replaced by other products making hunting less profitable (Ellis 2009). Thus, whaling gradually decreased from the 1960s onwards and it was officially banned in 1986 when the Moratorium of the International Whaling Commission ratified by Portugal in 1982 came into force. The last two sperm whales were hunted on Pico Island in 1987 setting the end of the whaling era in the Azores (Santos et al. 1995). Undoubtedly, whaling represented the first source of information about cetaceans in the Azores. Data obtained shed light on the distribution of sperm whales in space and time, their physical description and even their diet including deep-sea giant cephalopods (Clarke 1954, Clarke 1956, Clarke 1981). In 1987, the International Fund for Animal Welfare (IFAW), invited by the Azores Government and supported by the European Community, conducted feasibility studies to investigate the potential for whale watching in the Azores (Neves-Graça 2004). In 1989, the first activities started on Pico Island and they soon became a great attraction for tourists. Some of the lookouts working for the whalers joined the new business immediately, followed by some whalers as skippers, bringing their expertise and traditional ecological knowledge to a newer public (Neves-Graça 2006). In 1993, it was also established in São Miguel and, since then, whale watching has progressively extended to four of the archipelago's nine islands. Nowadays, 28 species of cetaceans have been listed around these islands (Silva et al. 2014, Azevedo and Barreiros 2019), making them one of the most recognised whale and dolphin watching destinations worldwide. In the Azores, this touristic activity mostly grew under an eco-tourism approach and, from the beginning, its regulation involved all stakeholders, including the government and the scientific community. In October 1998, the first “Bienal das Baleias”, held in Lajes do Pico, was a stage open for discussion, learning and training from multidisciplinary perspectives, looking for an “ecologically friendly” whale watching model (Neves-Graça 2004). As a consequence, the first whale watching legislation was published the following year (DLR 9/99/A) including an obligation to collect data on cetaceans sighted during the activity. This mandatory system proved ineffective and was soon abandoned, but it left a vacuum felt by all the parties involved: operators needed information to better plan a growing business and assess its sustainability, the government needed to monitor cetaceans to establish appropriate regulations and management plans and scientists saw the value of data collected cost-effectively, regularly and in the long-term. Discussions held during the last “Bienal das Baleias” in 2006, led to the development of the MONICET project: a consortium of a research centre and three whale watching companies, with economic support from the Azores Government, to establish a methodology to collect scientifically useful and reliable data compatible with the commercial operation and a database system to store, organise and disseminate the data on an open access basis.

## General description

### Purpose

We present the MONICET dataset of cetacean occurrences, based on opportunistic data collected by whale watching companies in the Azores. This dataset has already been published via GBIF, OBIS and EMODNET as a Darwin Core Archive. We aim to: (1) provide a detailed description of the methodology used for data collection; (2) acknowledge the limitations and potential sources of bias inherent to this dataset and (3) advise about the potential use of the data to minimise misinterpretation and maximise usability by external users.

## Project description

### Title

MONICET- the whale watching companies and the public at the service of knowledge and conservation of the Azores cetaceans

### Personnel

Project coordination was done by José M. N. Azevedo, Marc Fernández and Laura González García. Data presented here have been collected by people working for 11 of the 23 registered whale watching companies: Azores Experiences and Peter Whale Watch in Faial Island; Aqua Açores and Espaço Talassa in Pico Island; Futurismo Azores Adventures, Picos de Aventura, Terra Azul, Terra do Pico and Sea Colors in São Miguel Island and Atlantiangra, Ocean Emotion and Picos de Aventura in Terceira Island. Company participation is voluntary; MONICET is permanently open to receive data from any company.

### Study area description

**Figure 1.**
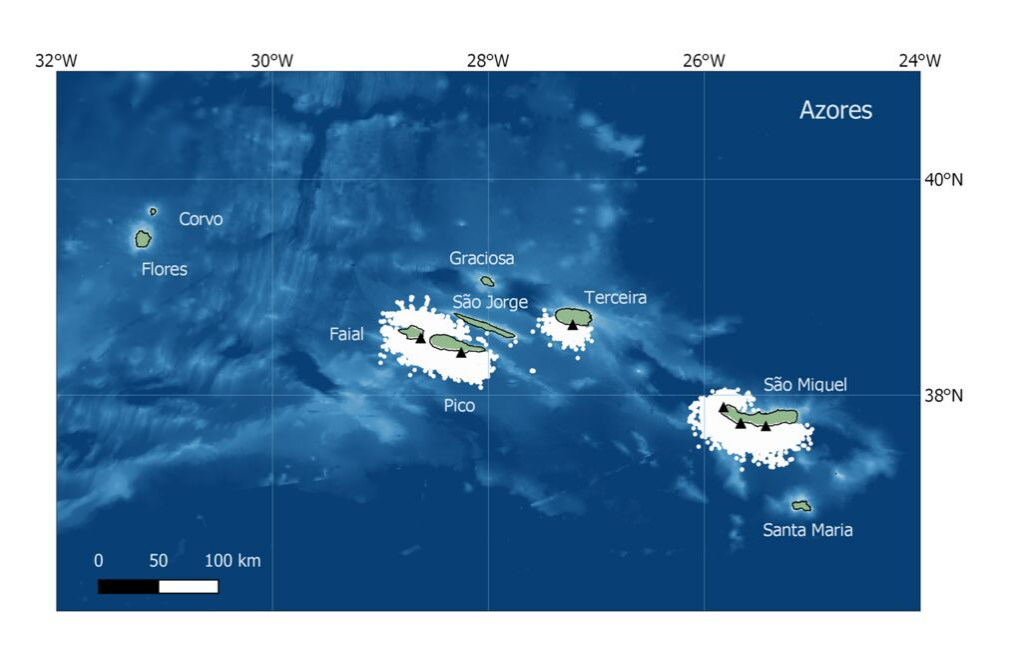
The Azores Archipelago, with the locations (white dots) of all the sightings recorded between 2009 and 2020 in the MONICET database. Base ports of contributing companies are indicated by black triangles.

The Azores Islands are located in the North Atlantic Ocean at 36-41° N and 24-32° W. The archipelago is composed of nine volcanic islands organised into three groups separated by deep waters (> 2000 m): the Western (Flores and Corvo), Central (Faial, Pico, Graciosa and Terceira) and Eastern groups (São Miguel and Santa Maria) (Fig. 1). The archipelago presents a well-defined oceanographic seasonality: March is usually the coldest month (SST ~ 15°C), while the highest temperatures are generally reached in September (SST ~ 25°C) (González García et al. 2022b). The spring bloom is noticeable each year by the increase in chlorophyll concentrations, which drastically drop to oligotrophic conditions during summer. The Azores are in a strategic location in the Mid-Atlantic, being affected by dynamic oceanographic features derived from the North Atlantic Current and the Azores Front/Current System, which enhance the primary production and the aggregation of life around these oceanic islands in comparison with surrounding waters (Caldeira and Reis 2017).

### Design description

MONICET is a collaborative platform to collect, organise and disseminate cetacean occurrence data and photo-identification images collected by whale watching companies in the Azores. Data are collected using a methodology developed collaboratively to provide scientifically useful information while remaining compatible with the commercial activity. An online database stores the data and a dedicated website (http://www.monicet.net/) provides a public interface to access basic geographical and statistical information. The project relies on the voluntary collaboration of the partners, mostly the whale watching companies and their staff, with the assumption that validated data will be openly shared and disseminated.

### Funding

MONICET received a starting grant in 2008 from the Azores Government (M5.2.2/I/ 005/2008, 'MONICET - As empresas e o público ao serviço do conhecimento e conservação dos cetáceos dos Açores'). Since then, its running costs have been supported by the University of the Azores and its research centres. It is currently hosted by the Institute of Marine Sciences - OKEANOS, financed through the FCT – Foundation for Science and Technology, I.P., under the project UIDB/05634/2020 and UIDP/05634/2020 and through the Regional Government of the Azores through the project M1.1.A/FUNC.UI&D/003/2021-2024. It was hosted until February 2023 by the Azorean Biodiversity Group, affiliated with the Centre for Ecology, Evolution and Environmental Changes (UID/BIA/00329/2020-2023 - Thematic Line 1 "Integrated Ecological Assessment of Environmental Change on Biodiversity; and PO Azores Project - M1.1.A/FUNC.UI&D/010/2021-2024). An ongoing grant from the PO Açores 2020 Programme (AÇORES 01-0145-FEDER-000079, 'MEEMO - Maintain, expand and exploit the cetacean observation platform MONICET. A unique opportunity for science, public policy and business') is dedicated to modernising the computer infrastructure and exploring the scientific value of the data. Additionally, over the years, many students financed by EU Programmes ERASMUS and EURODYSSÉE participated in data collection, digitalisation and validation.

## Sampling methods

### Study extent

Data were collected in the Azores Archipelago (Fig. 1) by whale watching operators located in the four islands where whale watching takes place: Faial, Pico, São Miguel and Terceira. The project started with three companies in São Miguel and reached a maximum of nine companies providing data simultaneously in 2019 (Table 1).

### Sampling description

Cetacean occurrence data were collected from January 2009 to December 2020 by whale watching companies running tours from the islands of Faial (Horta harbour), Pico (harbours of Lajes do Pico and Madalena), São Miguel (harbours of Ponta Delgada and Vila Franca do Campo) and Terceira (Angra do Heroismo harbour). Generally, whale watching tours last between 2.5 to 3 h and are conducted twice a day: one in the morning (9:00 h approx.) and the other in the afternoon (13:30 h approx.), with an exceptional third trip in the high season. In São Miguel, the activity operates year-round, while in the other islands, it is more seasonal, operating primarily between May and September.

On all the islands, cetaceans are first located from land by experienced observers working with powerful binoculars from strategic locations on the coast. Whale watching boats are then guided from land to the location of the cetaceans. Therefore, each trip often presents one or more sighting events, where boats normally slow down or stop for clients to watch the animals.

For the database presented here, qualified and trained guides on board (often biologists) register the data manually at sea and type the information into the online database once on land. The time of departure and arrival of the boat to the base port is noted. Once with the animals, time, location (read from a GPS receiver) and species are recorded as a minimum. In most cases, a location is registered when the boat arrives near the animals and another when it leaves. Occasionally, intermediate points are also recorded. If the boat does not stop, just one location is noted for that observation. Additionally, number of individuals, presence of different life stages (adults, juveniles or calves), behavioural state and association with other species could be recorded, as well as sea state and visibility.

### Quality control

The MONICET team provides annual training focused on data collection to the whale watching partners involved in the project. During these sessions, the desired data fields to be collected are extensively addressed and doubts about data collection are solved. The highly qualified profile of the guides or biologists of the companies, who are experienced and knowledgeable about cetaceans, is a reasonable guarantee of a correct species identification at sea.

Finally, information introduced in the online database is validated by a central team composed of researchers and trained interns. The validation process is mainly focused on mismatches in locations and timings and addresses any issues raised by the person entering the data. A dialogue is established, if necessary, until all the information is validated or the record is erased.

### Step description

The data have been published following the standardised format for biodiversity data of Darwin Core Archive (DwC-A). This model consists of a core data file (*eventTable*) that contains information about the location and time of the sampling events (the encounters), associated through common database keys (i.e. *eventID*), with two extension data tables: the *occurrenceTable*, which contains taxonomical information about each sighting and the Extended Measurement Or Fact *eMoFTable*, containing biological and environmental measurements.

A hierarchical system was considered where each whale watching trip is a *parent event* and each encounter belonging to that trip is an *event*. On each *event*, several occurrences may happen, considering an *occurrence* the sighting of a life stage of a species. For instance, a group of individuals may be recorded as an occurrence or separate occurrences can be recorded for each life stage (adult, juvenile, calf). Additionally, the sea state and the visibility, as well as the behaviour, the number of individuals sighted, the life stage and the sampling instrument name are related to the corresponding occurrences.

## Geographic coverage

### Description

Azores Archipelago, Portugal

### Coordinates

37.3118 and 39.908 Latitude; -25.008 and -28.9984 Longitude.

## Taxonomic coverage

### Description

Sightings were identified, whenever possible, to the species level. Otherwise, at least family was noted. Turtles were occasionally registered, although they were not the project's main goal. The dataset contains sightings of 22 species of cetaceans and three species of marine turtles (Table 2).

### Taxa included

**Table:** 

Rank	Scientific Name	Common Name
order	Cetartiodactyla	Cetaceans
order	Testudines	Sea turtles

## Temporal coverage

**Data range:** 2009-1-01 – 2020-12-31.

### Notes

Data were collected year-round in São Miguel and on a seasonal basis in Faial, Pico and Terceira.

## Usage licence

### Usage licence

Other

### IP rights notes

Creative Commons Attribution 4.0 Internacional

## Data resources

### Data package title

MONICET: Long-term cetacean monitoring in the Azores, based on whale watching observations (2009-2020).

### Resource link

https://doi.org/10.14284/599

### Alternative identifiers

https://www.vliz.be/imis?dasid=7948

### Number of data sets

1

### Data set 1.

#### Data set name

MONICET: Long-term cetacean monitoring in the Azores, based on whale watching observations (2009-2020).

#### Data format

Darwin Core Archive (DwC-A)

#### Character set

UTF-8

#### Download URL

https://mda.vliz.be/download.php?file=VLIZ_00000772_642fe989615ef444113174

#### Data format version

1.8.2

#### Description

The dataset includes all the records of cetaceans and turtles reported to the MONICET platform by whale watching companies in the Azores between 2009 and 2020 (Azevedo et al. 2023). It has been published as a Darwin Core Archive (DwCA), a standardised format for sharing biodiversity data as a set of one or more data tables. Data are available from OBIS, GBIF, EMODNET and IPT, all serving as a data repository. The core data table, the *eventTable* (Table 3), contains 37855 events, considering each observation as a separate *event* with a specific location and time. Each *event* belongs to a parentEvent corresponding to the whale watching trip in which it was recorded (in total, 8455 trips registered). The dataset also contains two extension tables linked to the core with the *eventID* field. The *occurrenceTable* (Table 4) contains 77572 occurrences, from which 374 are identified to family level and the rest correspond to 25 different species; and the *eMoFTable* (Table 5) contains 199792 measurements or facts related to each *event* (visibility or sea state) and/or to each *occurrence* (number of individuals, behaviour, life stage and sampling instrument).

**Data set 1.** 

Column label	Column description
parentEventID	Each whale watching trip.
eventID	Each encounter with cetacean or turtle observations.
occurrenceID	The species and respective life stage recorded.

## Additional information

Data collection for MONICET follows a well-defined protocol developed with several experts and the companies involved and widely shared. It standardises data collection and is compatible with the work onboard a whale watching vessel, while simultaneously recording scientifically useful information. Regular training sessions of company staff are conducted at least once a year, as this has proven to be effective to maintain company engagement and to enhance the long-term commitment from both sides.

The dataset provides ecological information about an extended group of cetacean species, including some whose global conservation status is a cause for concern, either Endangered (2 species), Near Threatend (2 species), Vulnerable (2 species) or even Data Deficient (1 species). The set of species convered by this database also corresponds to a wide range of ecological categories, from residents to seasonal visitors and others passing by in their annual migrations (Silva et al. 2014).

While the present dataset therefore constitutes a unique source of information on many aspects of cetacean biology and ecology, its sources of bias should be understood to minimise misuse and avoid misleading conclusions (Fernandez et al. 2018, Fernandez et al. 2021, González García et al. 2022a).

One of the main limitations of the dataset is the lack of absolute quantification of effort. In fact, whereas animals are mostly detected from land observations, the selection of what species to see (and therefore to record) is strongly influenced by commercial preferences. Boats will often prioritise shorter distances to the base port, locations with better sea conditions or even more appealing behaviour or species preferred by customers. For instance, a family of sperm whales or a playful group of dolphins would prevail over an elusive beaked whale or a diving whale away from the location of the boat. Notwithstanding, all companies follow approximately the same modus operandi, conducting trips of 2.5 to 3 h on average in the morning and in the afternoon and following the instructions given from land by specialised observers. At the same time, there is also a constant search effort by the boat crew at sea. No associated land observation recordings or boat tracks are available for this dataset. Both could be of interest to assess the scope and duration of land search and miles sailed by WW boats, which could be used as proxies for relative effort.

Species identification is assumed to be of high quality, given that the persons recording the data (the tour guides and boat skippers or interns supervised by them to assist on data collection) are highly experienced and often have Marine Biology degrees. However, precautions must be taken with particular taxa: (1) beaked whales (especially *Mesoplodon* spp.), whose species determination is difficult at sea due to their short time at the surface and often elusive behaviour; (2) Bryde’s whales, whose sighting frequency seems to be increasing, but may still be confused mainly with sei whales; and (3) the short-finned and long-finned pilot whales, which are difficult to differentiate at sea. Companies are instructed to record the predominant behavioural state at the time of arrival (foraging, resting, socialising and travelling). However, the behaviour at the boat's arrival can still be influenced by it or can result from the previous presence of other boats in the area (see, for the Azores, Visser et al. (2010), Cecchetti et al. (2017), Oliveira et al. (2022)). Additionally, sightings are often of short duration (a few minutes), so main behaviour is not always possible to assess in this short time. For instance, dolphins often interrupt their primary behaviour when the boat arrives and may only resume it when the boat leaves (e.g. dolphins stop travelling and bow-ride the boat for a while; thus, socialising instead of travelling will be recorded). Finally, caution is needed regarding the registered number of individuals. This generally corresponds to the individuals sighted from the boat, which does not necessarily agree with the number of individuals in the area. For instance, boats may record the number of dolphins of only a subgroup of a larger group of which they are not aware. Another common situation frequently happens with sperm whales, where independent individual sightings at a close distance and within a short time, may be registered instead of what was in fact one group of animals. This often happens, particularly with foraging behaviour (e.g. fluking). Additionally, data analyses should bear in mind that each sighting may be recorded by different boats simultaneously, as different companies work in the same area and often target the same encounters. The data presented here have been voluntarily collected manually, written on a paper at sea and typed into the database upon return to land, often not immediately and sometimes by another person. This process may lead to errors, particularly in geographical coordinates. A validation process, where a team with scientific supervision reviews every record, aims to minimise these errors, but inconsistencies may still be found.

From an academic perspective, MONICET data have been widely used by Biology students at the University of the Azores. Marine Mammals' classes are attended every year by dozens of students who actively explore the database and get hands-on experience with cetacean research and actual data. For the scientific community, the MONICET dataset’s fine-scale sampling, with long-term and regular cover in space and time in several islands of the archipelago, greatly complements traditional dedicated surveys, which often focus on more specific questions and covering shorter time periods. Insights that can be obtained with this kind of data include: (i) habitat preferences, (ii) seasonality, (iii) long-term changes or trends in distribution patterns or relative abundance, in correlation with oceanic variables or (iv) evidence of rare events, such as the presence of cryptic species, anomalous pigmentations or unusual types of behaviour.

Additionally, FAIR principles are considered to pursue better-quality scientific outputs (Wilkinson et al. 2016): (1) the dataset has associated metadata to facilitate computer reading and findability; (2) data have been published and made freely accessible online in different biodiversity repositories; (3) data have been standardised as far as possible following the international standards for biodiversity data to ensure interoperability; (4) biases and limitations are provided to minimise misinterpretation of the dataset and encourage proper use of it. Nonetheless, due to its opportunistic nature, caution is required when interpreting results derived from this database.

Over more than three decades of activity, whale watching has indeed evolved in the Azores, achieving high-quality benchmarks and successfully joining tourism, education and research. MONICET, together with all the partners and companies involved, has showcased its vast potential to maintain low-cost, long-term monitoring of cetaceans in the Azores. It has proved not only its scientific worth, but also the added value it brings for the whale watching companies and society at large.

## Figures and Tables

**Table 1.** Whale watching companies reporting data to MONICET per year.

**Island**	**Company**	**2009**	**2010**	**2011**	**2012**	**2013**	**2014**	**2015**	**2016**	**2017**	**2018**	**2019**	**2020**
Faial	Azores Experiences		X	X	X	X	X	X	X	X	X	X	
	Peter Whale Watch											X	
Pico	Aqua Açores									X	X	X	X
	Espaço Talassa											X	X
São Miguel	Futurismo Azores Adventure		X	X									
	Picos de Aventura	X	X	X	X	X	X	X	X	X	X	X	X
	Sea Colors							X	X	X	X	X	
	Terra Azul	X	X	X	X	X	X	X	X	X	X	X	X
	Terra do Pico										X	X	
Terceira	Atlantiangra										X		
	Ocean Emotion							X	X	X			
	Picos de Aventura									X	X	X	

**Table 2.** List of taxa recorded on the MONICET platform between 2009 and 2020. Higher-level classification follows the World Register of Marine Species (WoRMS Editorial Board 2023); IUCN global conservation status according to IUCN (2022); AphiaID is the unique and stable identifier associated with each taxon in the WoRMS database through the use of Life Science Identifiers (LSIDs).

Class	Order	Family	Scientific name	Common name	IUCN status	AphiaID
Mammalia	Cetartiodactyla	Balaenopteridae	Balaenopteridae n.i.	rorqual		136979
Mammalia	Cetartiodactyla	Balaenopteridae	*Balaenopteraacutorostrata*	minke whale	LC	137087
Mammalia	Cetartiodactyla	Balaenopteridae	*Balaenopteraborealis*	sei whale	EN	137088
Mammalia	Cetartiodactyla	Balaenopteridae	*Balaenopteraedeni*	Bryde's whale	LC	137089
Mammalia	Cetartiodactyla	Balaenopteridae	*Balaenopteramusculus*	blue whale	EN	137090
Mammalia	Cetartiodactyla	Balaenopteridae	*Balaenopteraphysalus*	fin whale	VU	137091
Mammalia	Cetartiodactyla	Balaenopteridae	*Megapteranovaeangliae*	humpback whale	LC	137092
Mammalia	Cetartiodactyla	Delphinidae	Delphinidae n.i.	dolphins		136980
Mammalia	Cetartiodactyla	Delphinidae	*Delphinusdelphis*	common dolphin	LC	137094
Mammalia	Cetartiodactyla	Delphinidae	*Globicephalamacrorhynchus*	short-finned pilot whale	LC	137096
Mammalia	Cetartiodactyla	Delphinidae	*Globicephalamelas*	long-finned pilot whale	LC	137097
Mammalia	Cetartiodactyla	Delphinidae	*Grampusgriseus*	Risso's dolphin	LC	137098
Mammalia	Cetartiodactyla	Delphinidae	*Orcinusorca*	orca	DD	137102
Mammalia	Cetartiodactyla	Delphinidae	*Pseudorcacrassidens*	false killer whale	NT	137104
Mammalia	Cetartiodactyla	Delphinidae	*Stenellacoeruleoalba*	striped dolphin	LC	137107
Mammalia	Cetartiodactyla	Delphinidae	*Stenellafrontalis*	Atlantic spotted dolphin	LC	137108
Mammalia	Cetartiodactyla	Delphinidae	*Tursiopstruncatus*	bottlenose dolphin	LC	137111
Mammalia	Cetartiodactyla	Kogiidae	Kogiidae n.i.	kogiidae		136982
Mammalia	Cetartiodactyla	Kogiidae	*Kogiabreviceps*	pygmy sperm whale	LC	137113
Mammalia	Cetartiodactyla	Physeteridae	*Physetermacrocephalus*	sperm whale	VU	137119
Mammalia	Cetartiodactyla	Ziphiidae	Ziphiidae n.i.	beaked whale		136986
Mammalia	Cetartiodactyla	Ziphiidae	*Hyperoodonampullatus*	North Atlantic bottlenose whale	NT	343899
Mammalia	Cetartiodactyla	Ziphiidae	*Mesoplodonbidens*	Sowerby's beaked whale	LC	137121
Mammalia	Cetartiodactyla	Ziphiidae	*Mesoplodondensirostris*	Blainville's beaked whale	LC	137122
Mammalia	Cetartiodactyla	Ziphiidae	*Mesoplodonmirus*	True's beaked whale	LC	137126
Mammalia	Cetartiodactyla	Ziphiidae	*Ziphiuscavirostris*	Cuvier's beaked whale	LC	137127
Reptilia	Testudines	Cheloniidae	Cheloniidae n.i.	cheloniid		987094
Reptilia	Testudines	Cheloniidae	*Carettacaretta*	loggerhead turtle	VU	137205
Reptilia	Testudines	Cheloniidae	*Cheloniamydas*	green turtle	EN	137206
Reptilia	Testudines	Dermochelyidae	*Dermochelyscoriacea*	leatherback turtle	VU	137209

**Table 3.** Description of variables in the *eventTable*.

**Column label**	**Column description**
type	Refers to the type of sampling: 'cruise' (for each whale-watching trip) or 'sample' (for each encounter)
institutionCode	University of the Azores, whose acronym is 'UAc'.
datasetName	All records are derived from the 'MONICET' dataset.
eventID	Unique identifier for each encounter (i.e. sampling event) in the dataset. Composed of the 'parentEventID' + the number of the encounter (as 'encounter00000').
parentEventID	A unique identifier that groups potentially several events. In this case, each whale-watching trip is considered a parent event. The ID is composed by 'institutionCode' + 'datasetName' + number of whale-watching trip (as 'cruise0000').
samplingProtocol	All the encounters were recorded as 'visual observation from whale watching boat'.
eventDate	Date and time, or time interval when available, when an encounter was registered. Formatted as 'YYYY-MM-DDT00:00/YYYY-MM-DDT00:00'.
minimumDepthInMetres	Minimum depth at which the sighting was recorded (always 0 m, as animals are observed on the surface).
maximumDepthInMetres	Maximum depth at which the sighting was recorded (always 0 m, as animals are observed on the surface).
decimalLatitude	The latitude (in decimal degrees, using the spatial reference system in geodeticDatum) of the location of the encounter.
decimalLongitude	The longitude (in decimal degrees, using the spatial reference system in geodeticDatum) of the location of the encounter.
geodeticDatum	The ellipsoid, geodetic datum or spatial reference system (SRS) upon which the geographic coordinates given in decimalLatitude and decimalLongitude are based.
footprintWKT	A Well-Known Text (WKT) defines the location. It includes all the locations assigned to an event: a multipoint string for all the locations of a whale-watching trip (cruise); a line string or a point for the location(s) of an encounter.

**Table 4.** Description of the variables in the *occurrenceTable*.

**Column label**	**Column description**
collectionCode	Abbreviation of the dataset name, in this case, *MONICET*.
basisOfRecord	The specific nature of the data record, in this case, all as *HumanObservation*.
occurrenceID	Unique identifier for each *occurrence* within an *event* in the dataset. Composed of *eventID* + the 3-letter code for the species and APHIA-ID + life stage (adult, juvenile or calf).
recordedBy	Name of the whale watching company contributing the information.
occurrenceStatus	Statement about the presence or absence of a taxon at the specified location. All the *occurrenceStatus* in this database are *present*.
eventID	Unique identifier for each encounter or sampling event in the dataset. Composed of the *parentEventID* + the number of the encounter (as 'encounter00000').
scientificNameID	Life Sciences Identifier (LSID) assigned to the taxon by WoRMS.
scientificName	The full scientific name.
scientificNameAuthorship	The authorship information for the *scientificName* formatted according to the conventions of the applicable nomenclatural code.

**Table 5.** Description of the variables in the ‘eMoFTable’.

**Column label**	**Column description**
measurementID	Unique identifier for the MeasurementOrFact (information on measurements, facts, characteristics or assertions). Composed of *eventID* + *measurementType*.
occurrenceID	Unique identifier for each occurrence within an event. Composed of *eventID* + the 3-letter code for the species and AphiaID + life stage (adult, juvenile or calf). The same encounter can include different occurrences of the same species, one per life stage.
measurementType	The nature of the measurement, fact, characteristic or assertion. The options available in this dataset are *Beaufort wind force*, *behaviour*, *individualCount* (best estimate), *life stage* (adult, juvenile or calf), *sampling instrument name* and *visibility*.
measurementTypeID	Definition according to the NERC Vocabulary Server (NVS) managed by the British Oceanographic Data Centre (BODC).
measurementValue	The value of the measurement, fact, characteristic or assertion. In this dataset: level of *Beaufort wind force*; *behaviour* (foraging, resting, socialising, travelling); *individualCount* (best estimate) (number of individuals sighted); and *visibility* (0-1000 m, 1000-5000 m, 5000-10000 m, >10000 m).
measurementValueID	British Oceanographic Data Centre BODC codes for the measurement values.
measurementUnit	The units associated with *measurementValue*.
measurementUnitID	British Oceanographic Data Centre BODC codes for the measurement units.
